# Sedimentary and geochemical characteristics of Triassic new type of polyhalite potassium resources in Northeast Sichuan and its genetic study

**DOI:** 10.1038/s41598-020-69063-2

**Published:** 2020-08-11

**Authors:** Jiaai Zhong, Mianping Zheng, Yongsheng Zhang, Xueyuan Tang, XueFei Zhang

**Affiliations:** 1 Key Laboratory of Metallogeny and Mineral Assessment, Ministry of Natural Resources, Beijing, 100037 China; 2 Institute of Mineral Resources Chinese Academy of Geological Sciences, Beijing, 100037 China; 3 Key Laboratory of Saline Lake Resources and Environment, Ministry of Natural Resources, Beijing, 100037 China; 4 Sichuan geology and mineral exploration and Development Bureau of four O five geological team, Dujiangyan, 611830 Sichuan China

**Keywords:** Solid Earth sciences, Chemistry

## Abstract

Polyhalite has been discovered for years in the Triassic of the Sichuan Basin. However, it is difficult to exploit and utilize such polyhalite because of its deep burial depth and its coexistence with anhydrite or dolomite. Therefore, it has always been regarded as “dead ore”. Based on slice identification, X-powder diffraction, chemical analysis, REEs analysis and strontium isotope test on halite samples from the fourth and fifth member of Jialingjiang Formation to Leikoupo Formation of Wells ZK601 and ZK001 in Xuanhan area, Northeast Sichuan Basin, this paper discovers thick layers of granular polyhalite associated with halite and the polyhalite content accounts for 10–30%. These deep polyhalites can be obtained by water-soluble mining and utilized, so they are called “new type polyhalite potash deposits”. The deposit is deep buried at 3,000 m underground, and the thickness of a single layer can be more than 30 m. It is stable in regional distribution. The discovery of the “new type polyhalite potash deposits” has “activated” polyhalite, which has been considered as deep “dead ore” and has great significance for potash prospecting in China.

## Introduction

China’s marine potassium is mainly developed in the Lower-Middle Triassic Jialingjiang Formation and the Leikoupo Formation of the Sichuan Basin, and abundant potassium salts such as polyhalite and potassium-rich brine have been found^1–7^. In-depth research has been conducted for the Triassic polyhalite in the Sichuan Basin, and the research covers the formation conditions^8,9^, the genesis mechanism^10–12^ , resource and reserve evaluation, and comprehensive development and utilization. The resource evaluation and exploitation of the shallow-layer polyhalite in the Nongle Village, Qu County, East Sichuan has achieved good results^13–15^. However, the research has been concentrated on the bedded, stratoid, lenticular polyhalite occurring in the anhydrite or interbedding with anhydrite in different thickness. This kind of polyhalite does not coexist with halite, shown in the section, and is mostly deep buried (2,000–5,000 m underground). Since it is difficult to be utilized, it has always been considered as “dead ore”. Sichuan BestRed Mining Company has made some progress in in-situ dissolution experiment of deep polyhalite in Guang'an, Sichuan. However, there is still a long way to go to achieve low-cost and efficient industrial mining.

Wang and Zheng^11^ found polyhalite in Triassic rock salt in Changshou area, East Sichuan. Afterwards, with the support of the National Geological Survey, the team led by Zheng Mianping conducted in-depth research on the core and well logging data of the potash exploration wells in the Puguang area of Xuanhan County, Sichuan Province and cooperated with Hengcheng company, 405 Geological Brigade, Chengdu University of Technology and other relevant units to form the synergy effect of innovation. Their findings show that thick layers of granular polyhalite associated with halite are encountered by boreholes such as Hengcheng 2, Hengcheng 3, ZK601, and ZK001, and polyhalite particles of different sizes are distributed in halite matrix deeper than 3,000 m.

Due to the solubility of halite, this kind of deep granular halite can be directly extracted by water-soluble mining and utilized. Academician Zheng Mianping called it “polyhalite grain cemented by halite crystal”—a new type of polyhalite potash deposits (PPD). At present, there are relatively few studies on the characteristics and genesis of the PPD from the fourth and fifth member of Jialingjiang Formation to Leikoupo Formation of the Triassic in the Xuanhan area, and there is a lack of research on its distribution law and prospects for potassium formation. In this paper, the halite samples from the fourth and fifth member of Jialingjiang Formation to the first member of Leikoupo Formation of potash exploration Wells ZK601 and ZK001 in Xuanhan area are systematically collected, and the slice identification, X-powder diffraction, chemical analysis, REEs analysis and strontium isotope test are carried out. The characteristics and genesis of the PPD are studied. At the same time, the well logging data of more than 30 natural gas wells in this area are interpreted, the “new type of polyhalite potash deposits” is identified and its distribution law is studied. Based on this, the prospect of potash in this area is analyzed in order to open up a new direction for the exploration of marine potash in China.

## Geological background

**Figure 1:**
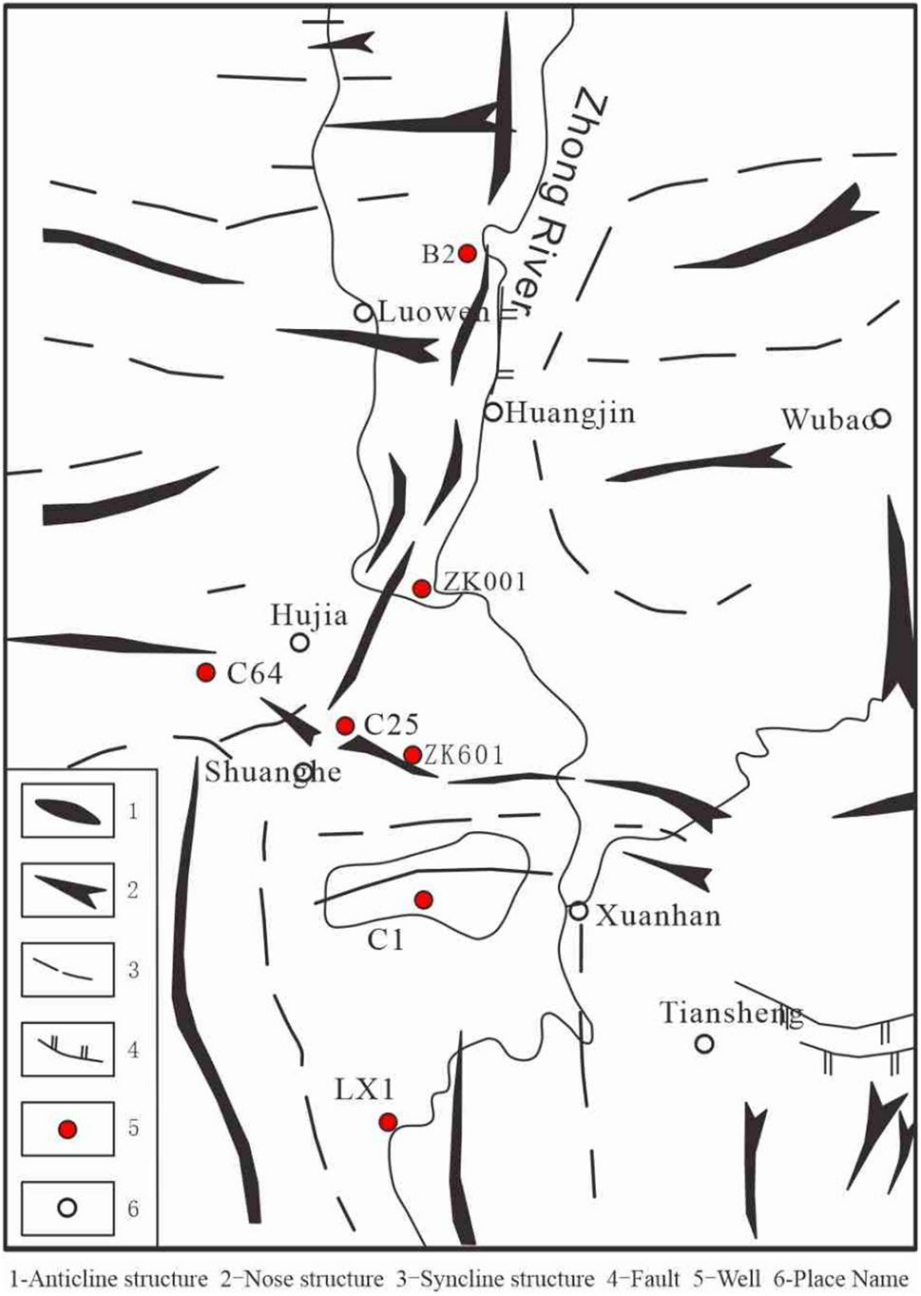
Structural location map of study area. Map generated using Geomap4.0 (https://www.jurassic.com.cn/zh-cn/Products/Service/145).

Puguang gas field is located in the northeast of Sichuan Basin. Tectonically, it belongs to the northwest of the upper Yangtze Block, the hinterland of Sichuan foreland basin, and lies in the transitional zone between the front fault-fold belt of the Dabashan nappe belt and the arcuate fault-fold belt of East Sichuan. The Huangjinkou anticline is narrow and asymmetric and is slightly box shaped, with its axis trending NE 50°–60°. The dip angle of the south east wing is 30°–60°, and that of the north west wing is 20°–35° (Fig. 1).

The strata in the study area are a set of carbonate rock and clastic rock formations of shallow marine and coastal facies, with no magmatism and metamorphism. In this area, the Lower and Middle Jurassic Ziliujing Formation and the strata below are all buried in the hinterland. The salt bearing strata are located in the fourth and fifth members of the Jialingjiang Formation and the first member of Leikoupo Formation of the Triassic in Xuanhan area, with burial depth of about 3000 m. The salt bearing strata in the study area are a set of evaporites (carbonate–sulfate–chloride combination), which is mainly composed of dolomite, calcareous dolomite, dolomitic limestone, anhydrite and halite, mixed with polyhalite. A small amount of magnesite is seen, and the cumulative thickness is about 300 m.

## Results

### Profile of salt bearing series

**Figure 2:**
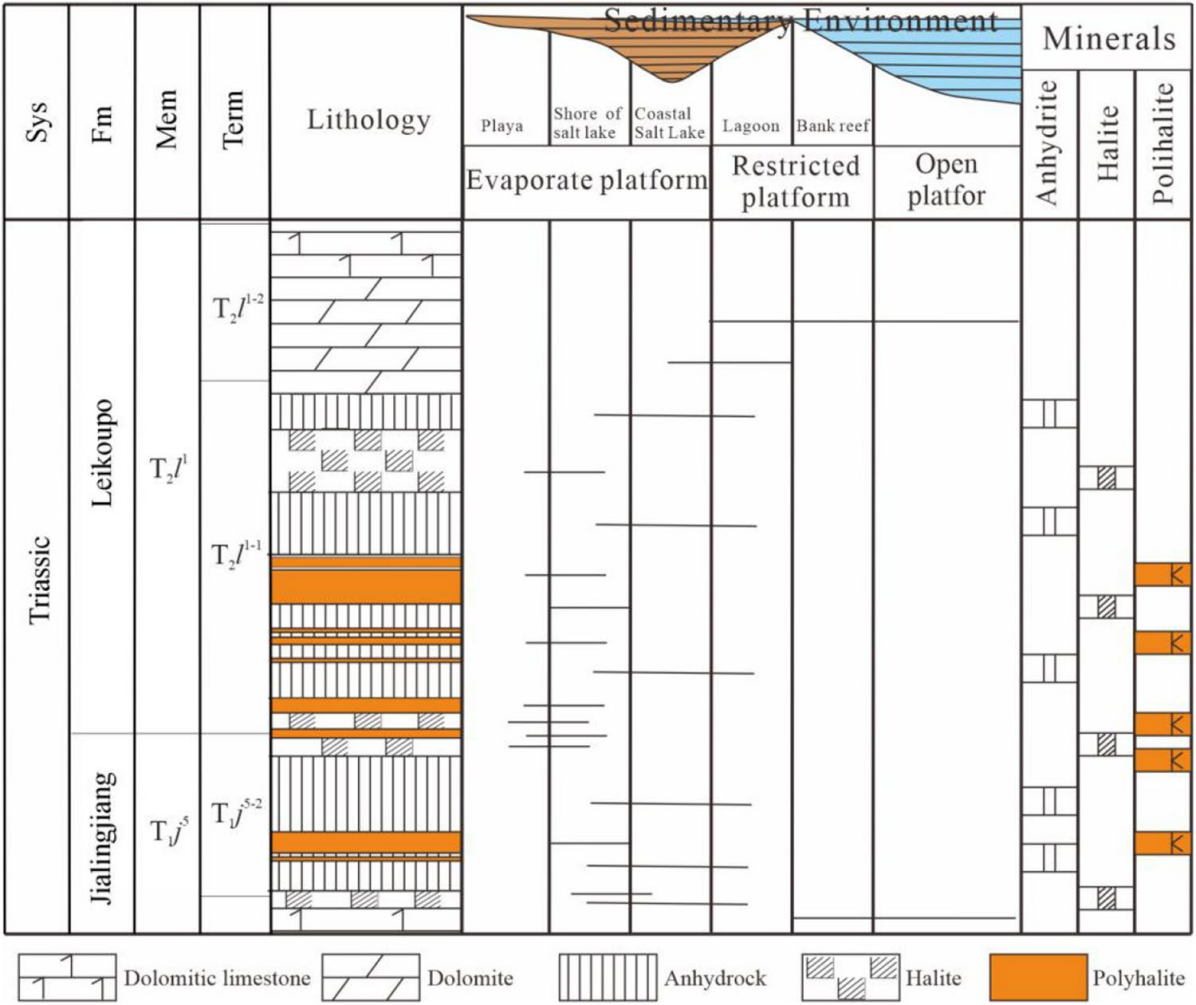
Profile of polyhalite type potash beds in ZK601.

The PPD was developed in the salt forming period of T_1_*j*^5^-T_2_*l*^1-1^. The salt bearing profile consists of carbonate rock, sulfate rock, chloride rock (containing polyhalite), sulfate rock and carbonate rock from the bottom to the top, forming a complete salt bearing profile (Fig. 2). It reflects the deposition process of the salt formation from desalination to salinization, and then gradually desalination. Salt bearing series was developed completely, which is conducive to the preservation of soluble salts.

### Cross section characteristics of PPD

According to the results of core drilling and logging, combined with the results from identification of salt bearing series through formation logging, this paper identifies several new type polyhalite potash beds encountered by potash exploration wells and oil and gas drilling wells in the study area and draws three well tie profiles perpendicular to and three ones parallel to the Huangjinkou anticline to depict the distribution of PPD in the study area.

**Figure 3:**
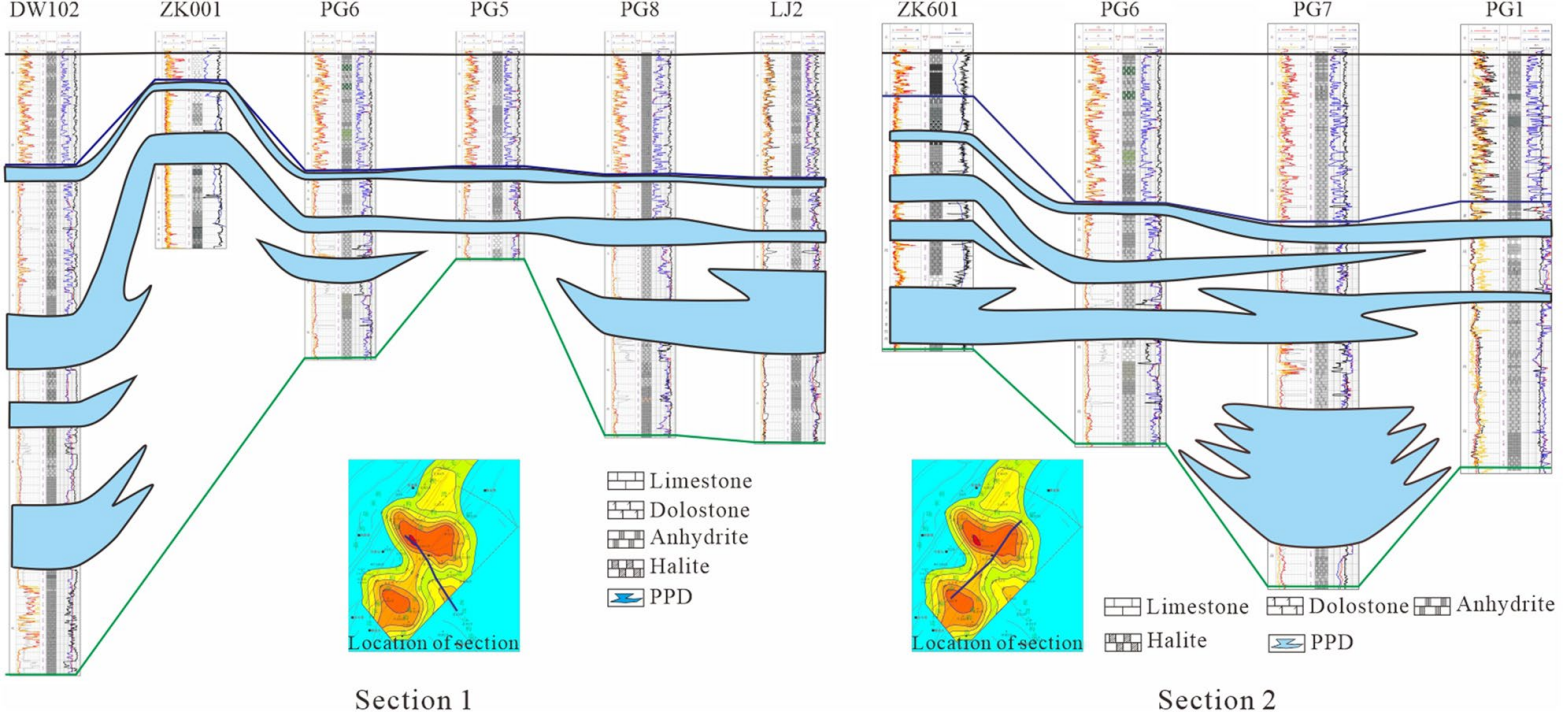
Well tie profiles of polyhalite bearing halite in the Huangjinkou anticline in Northeast Sichuan Basin.

The NW–SE trending section 1 passes through Wells Dawan 102, ZK001, Puguang 6, Puguang 5, Puguang 8 and Laojun 2 (Fig. 3) and is perpendicular to the axis of Huangjinkou anticline. Section 1 is in the middle of Huangjinkou anticline and it passes through the core and southeast wing of the anticline. In this section, the thickness of the first member of Leikoupo formation is basically stable, and the fourth and fifth member of Jialingjiang Formation shows obvious thickening anomaly in the core of anticline, which is caused by the compression and deflection of gypsum salt layer. The thickness of PPD decreases first and then increases from the core to the southeast wing of the anticline. The PPD at the top of T_1_*j*^5^ of each well is relatively stable. Multi-layer PPD is encountered by the lower part of Well Dawan-102 in the core of anticline. The PPD in the middle of Well Laojun 2 at the SE end of the section is thicker than that of other wells. The SW-NE trending section 2 passes through Wells ZK601, Puguang 6, Puguang 7 and Puguang 1 (Fig. 3). Section 2 is parallel to the axis of Huangjinkou anticline and is located at its southeast wing. In this section, the thickness of T_2_*l*^1^ and T_1_*j*^4 + 5^ increases first and then decreases from south to north, and it is slightly thick in Well Puguang-7 in the middle and is small in Well ZK601 in the south because T_1_*j*^4^ is not drilled through. In this section, the PPD is relatively developed, and its thickness decreases then increases and decreases again from south to north. There are two sedimentary centers in both Well ZK601 and Well Puguang 7.

**Figure 4:**
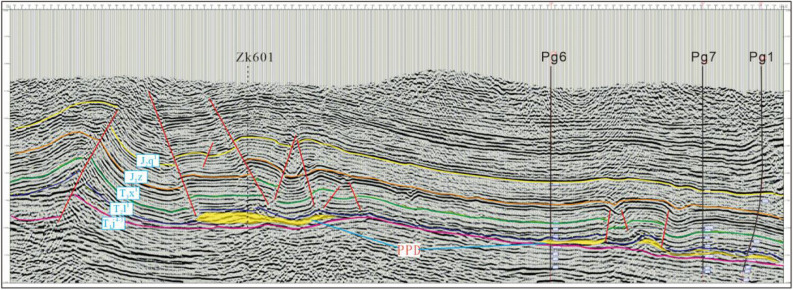
Seismic and geological profile of Huangjinkou anticline in Northeast Sichuan Basin.

At the same time, the seismic and geological profile parallel to the core of the anticline is drawn through Wells ZK601, Pg 6, Pg 7 and Pg 1 (Fig. 4). The profile shows that the thickness of T_2_*l*^1^-T_1_*j*^4+5^ of Huangjinkou anticline is structurally controlled, and the formation in the core of the anticline is obviously thickened. The thickness change of PPD is consistent with that of T_2_*l*^1^-T_1_*j*^4+5^ .

### Plane distribution of the PPD

**Figure 5:**
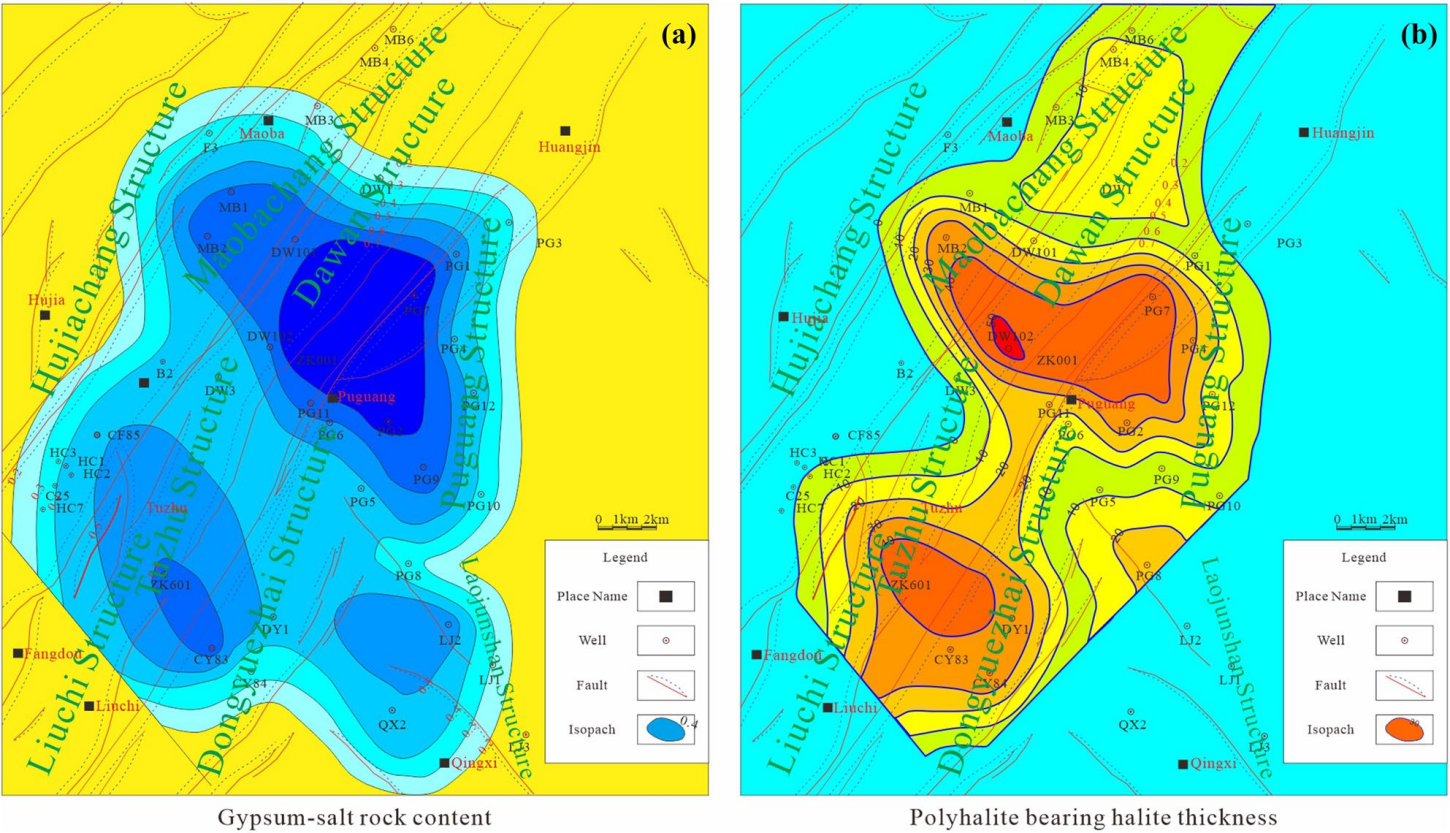
(**a**) the ratio of gypsum salt rock thickness to formation thickness of T_2_*l*^1^-T_1_*j*^4+5^ of Huangjinkou anticline in Northeast Sichuan Basin; (**b**) plane distribution of PPD thickness of T_2_*l*^1^-T_1_*j*^4+5^ of Huangjinkou anticline in Northeast Sichuan Basin. Map generated using Geomap4.0 (https://www.jurassic.com.cn/zh-cn/Products/Service/145).

According to the logging results of potash exploration wells and oil and gas wells in Huangjinkou anticline, the thickness data of gypsum salt rock and the thickness of PPD from the fourth member of Jialingjiang Formation to the first member of Leikoupo Formation in more than 30 wells were well collected, and the ratio of gypsum salt rock thickness to formation thickness was calculated. On this basis, the plane distribution showing the ratio of gypsum salt rock thickness to formation thickness of T_2_*l*^1^-T_1_*j*^4 + 5^ of Huangjinkou anticline in Northeast Sichuan Basin (Fig. 5a) and plane distribution of PPD thickness (Fig. 5b) were plotted, and the research on the distribution law of PDD was conducted.

Partly, the ratio of gypsum salt rock thickness to formation thickness reflects the paleosedimentary environment, and the plane distribution of gypsum salt rock thickness indicates the paleoevaporation center. The thickness of gypsum salt rock of T_2_*l*^1^-T_1_*j*^4 + 5^ in the study area is between 31 and 378 m and is mainly in the range of 100–200 m. The ratio of gypsum salt rock thickness to formation thickness is 0.22–0.74, and three high value centers can be seen in the plane distribution, showing local increase and obvious lateral change. There is a certain correlation between Fig. 5a and Fig. 5b. The positions of three high-value centers in Fig. 5a are basically consistent with those in Fig. 5b. The three centers are Well ZK601 of Tuzhu-Liuchi structure in the southwest section of Huangjinkou anticline, Well102 of Maoba-Dawan structure in the northeast section and Well Puguang 8 of Laojunshan structure in the southeast wing of Huangjinkou anticline. The PPD thickness is between 1.3 m (Well Dawan 3) and 84.7 m (Well Dawan 102), and it changes obviously along the anticline dip, showing that it is controlled by the ancient evaporation center and structure.

### Structure and mineral characteristics of PPD

**Figure 6:**
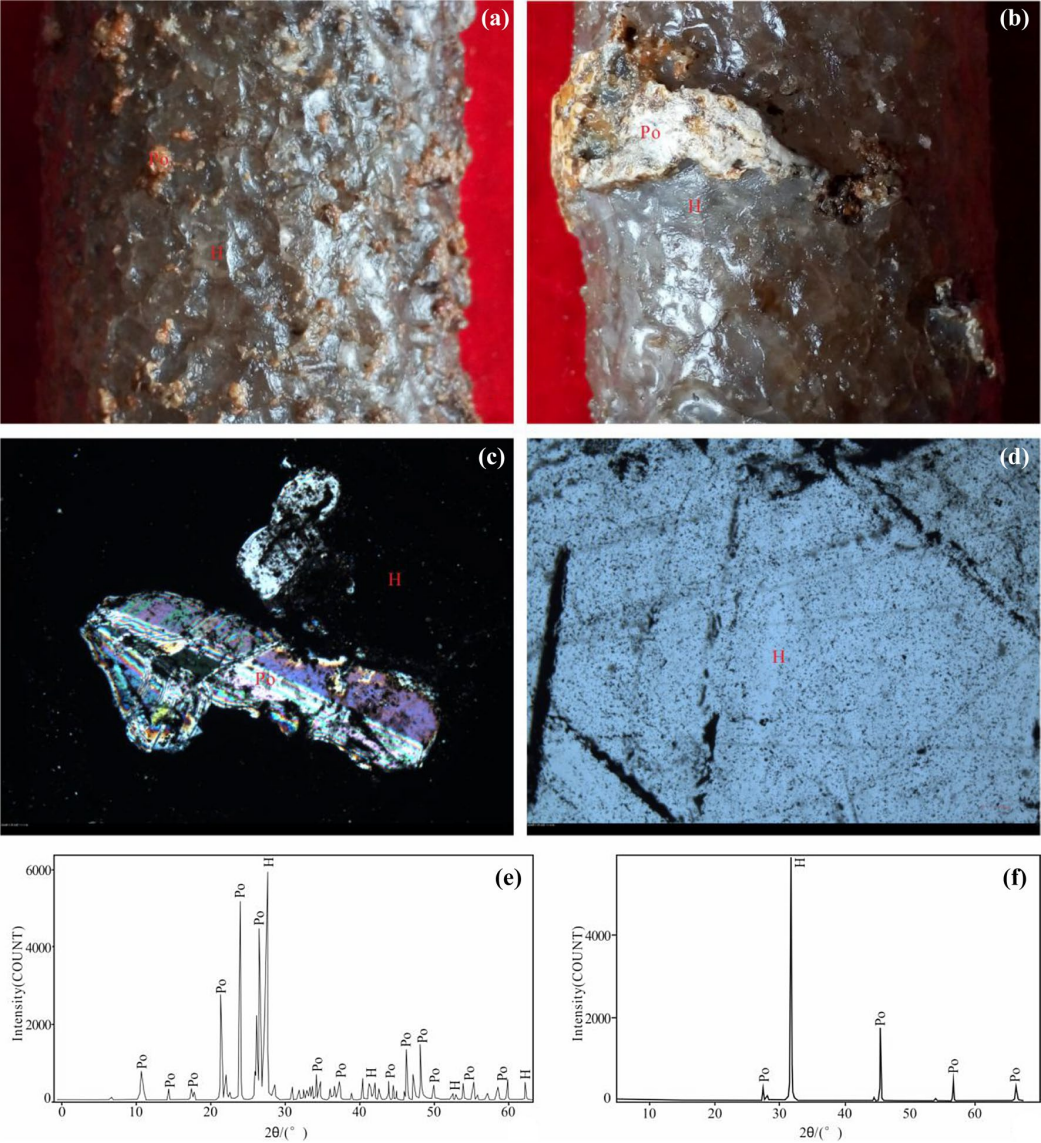
Core section photograph, microscopic photograph and XRD graph of PPD. (**a**,**b**) Photo of core section of polyhalite bearing halite; (**c**) microscopic photograph of polyhalite bearing halite, perpendicular polarized light 10 × 5; (**d**) microscopic photograph of polyhalite bearing halite, plane polarized light 10 × 5; (**e**,**f**) XRD graph of polyhalite bearing halite.

The PPD is dominated by halite and polyhalite, with a small amount of anhydrite and magnesite. On the core, since more insoluble than halite, the polyhalite occurs as granular particles protruding on the surface of halite. In addition, due to the influence of drilling mud, the polyhalite is yellow–brown and thus easy to be identified. The matrix of PDD consists of light gray transparent-translucent halites, which dominated by medium-coarse crystal texture. The silt- to boulder-sized polyhalite particles are scattered in the matrix. The polyhalites are mainly granular (Fig. 6a), with a few agglomerate and stratoid (Fig. 6b). Polyhalites are mostly micro-fine-grained under the electron microscope. They are colorless, transparent, subhedral-xenomorphic granular, tabular, striped, and bedded (locally assembled ones), and are low relief in the systems of monopolarizer. The interference color order of these crystals are up to second blue, and the polysynthetic twins are common (Fig. 6c, d) under perpendicular polarized light.

In order to further clarify the types of minerals, X-ray powder diffraction (XRD) was used. The results of XRD analysis show that the sample is mainly composed of halite and polyhalite, and the content of halite and polyhalite in each sample varies, as shown in Fig. 6e,f.

### Chemical composition of PPD

A part of PPD samples from Wells ZK601 and ZK001 were analyzed (Table 1). The K^+^ content is between 0.79% and 4.37%, with an average value of 1.79%. Some samples have the industrial grade of potassium. The Cl^–^ content is 34.78–67.32%, with an average value of 53.95%. The Br^–^ content is 52 × 10^–6^ to 276 × 10^–6^, with an average value of 170 × 10^–6^. The ratio of Br × 10^3^/Cl is 0.11–0.47, with an average value of 0.32.

**Table 1:** Chemical composition of polyhalite bearing halite samples.

Sample number	Na^+^ (%)	K^+^ (%)	Ca^2+^ (%)	Mg^2+^ (%)	Cl^−^ (%)	SO_4_^2−^ (%)	Br^−^ (10^–6^)	Br × 10^3^/Cl
ZK601-01	33.78	1.14	3.95	0.37	39.16	11.44	121	0.31
ZK601-02	29.52	0.79	9.50	1.60	34.78	25.60	162	0.47
ZK601-07	17.15	1.79	4.39	0.53	58.36	17.77	193	0.33
ZK601-08	17.04	1.87	4.90	0.57	56.19	19.42	163	0.29
ZK601-09	17.78	1.70	4.42	0.53	58.43	17.12	181	0.31
ZK601-10	18.74	1.37	4.11	0.45	59.78	15.52	209	0.35
ZK601-11	18.71	1.96	3.22	0.61	59.98	15.50	192	0.32
ZK601-12	21.09	1.25	2.62	0.39	63.65	10.99	255	0.40
ZK601-13	20.53	1.07	1.87	0.32	67.32	8.89	276	0.41
ZK601-23	23.49	4.37	5.97	1.50	39.91	24.76	156	0.39
ZK001-3	37.08	1.14	0.89	0.17	61.02	0.77	192	0.31
ZK001-4	34.31	1.55	1.66	0.54	55.89	6.03	147	0.26
ZK001-5	30.56	2.48	2.79	0.83	51.83	11.45	161	0.31
ZK001-6	33.45	1.47	2.25	0.64	56.92	5.27	132	0.23
ZK001-8	34.41	1.10	1.77	0.43	57.80	4.51	202	0.35
ZK001-9	31.36	2.83	2.68	0.88	49.47	12.82	52	0.11
ZK001-12	28.14	2.55	4.91	1.08	46.63	16.79	96	0.21

### The REE characteristics of a PPD

The REE analysis results of some samples from Well ZK601 (Table 2) shows that the ∑REE is low, ranging from 9.24 to 63.54 μg/g, with an average of 34.17 μg/g. The ∑REE of samples is far lower than the average ∑REE of PAAS (184.77 μg/g), indicating that the PPD is rarely affected by terrigenous material in the process of deposition and diagenesis.

**Table 2:** REE data of the samples in Well ZK601.

Sample number	La	Ce	Pr	Nd	Sm	Eu	Gd	Tb	Dy	Ho	Er	Tm	Yb	Lu	ΣREE	LREE/HREE	(La/Sm)_N_	(Gd/Yb)_N_	δEu	δCe
H6	1.61	1.44	1.31	0.83	0.62	0.41	0.46	0.64	0.31	0.42	0.33	0.31	0.24	0.31	9.24	2.06	2.62	1.94	0.76	0.98
H7	3.55	3.02	2.54	2.00	1.23	0.82	0.89	0.85	0.62	0.56	0.62	0.62	0.57	0.62	18.51	2.46	2.88	1.55	0.77	0.99
H8	2.58	2.48	2.30	1.67	1.18	0.68	0.66	0.85	0.47	0.56	0.48	0.62	0.48	0.62	15.60	2.30	2.19	1.37	0.74	1.02
H9	1.61	1.62	1.39	1.00	0.82	0.41	0.42	0.43	0.31	0.42	0.33	0.31	0.33	0.31	9.72	2.39	1.97	1.27	0.66	1.08
H11	2.26	1.73	1.39	1.00	0.56	0.68	0.39	0.43	0.25	0.28	0.29	0.31	0.29	0.31	10.16	3.01	4.00	1.34	1.43	0.95
H12	5.16	3.91	2.87	1.83	0.92	0.54	0.97	1.06	0.84	0.84	0.86	0.93	0.91	0.93	22.57	2.08	5.59	1.06	0.58	0.97
H14	13.23	11.42	8.77	6.83	4.31	1.50	2.74	2.55	2.33	2.23	2.00	1.85	1.91	1.86	63.54	2.63	3.07	1.43	0.42	1.04
H17	9.35	8.03	6.64	5.33	3.18	1.36	1.81	1.49	1.15	1.11	1.14	1.23	1.24	1.24	44.33	3.25	2.94	1.46	0.54	1.00
H21	2.90	2.24	1.72	1.17	0.62	0.41	0.31	0.21	0.22	0.28	0.24	0.31	0.19	0.31	11.12	4.38	4.72	1.61	0.88	0.97
H22	7.10	5.63	5.08	3.33	1.69	0.54	1.00	0.85	0.84	0.70	0.67	0.62	0.62	0.62	29.30	3.95	4.19	1.61	0.40	0.92

The REE distribution model can be used to determine the source of diagenetic fluid^16^. The REE distribution model of sea water is characterized by enrichment of LREE and relative loss of HREE^17^. The values of ΣLREE/ΣHREE of samples range from 2.06 to 4.38, with an average of 3.26, showing the characteristics of distinct REE differentiation, LREE enrichment and HREE depletion, and this is similar to the case in seawater. (La/Sm) _n_ reflects the fractionation degree of LREE elements, and (Gd/Yb)_n_ reflects the fractionation degree of HREE. The (La/Sm)_n_ ranges from 1.97 to 5.59, with an average of 4.10. The (Gd/Yb)_n_ ranges from 1.06 to 1.94, with an average of 1.43. This shows that the fractionation is moderate for LREE, while not obvious for HREE, reflecting the deep water environment during deposition.

The REE are variable valence elements, especially Ce and Eu, which are prone to change in valence state in different redox environments^18^. In the oxidation environment, Ce^3+^ in water is easily oxidized to Ce^4+^, resulting in the negative abnormality of Ce in sediment (δCe < 1). On the contrary, in the reduction environment, Ce^3+^ concentration in water increases, resulting in the positive anomaly of Ce in sediments^19^. In the hydrothermal fluid, the main form of Eu is Eu^2+^ under the condition of high temperature and reduction. When the hydrothermal fluid acts on the sediments, it will lead to the positive anomaly of Eu (δEu > 1)^20^. Therefore, the Ce and Eu contents in sedimentary rocks can be used to judge the redox condition of water body during sedimentation and whether there was transforming effect by deep fluids in the later stage of diagenesis. The δCe value of the PDD in Well ZK601 is between 0.92 and 1.08, with an average of 0.98, showing weak negative anomaly and reflecting the reduction environment during deposition; the δEu value is between 0.40 and 1.43, with an average of 0.56, showing a medium loss and indicating that there is no influence from hydrothermal fluids during deposition and diagenesis.

### Strontium isotope characteristics of PPD

**Figure 7:**
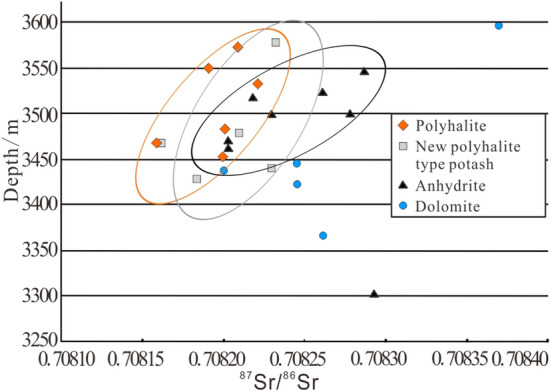
^87^Sr/^86^Sr of different kinds of lithologies in well ZK601.

The ^87^Sr/^86^Sr ration of seawater is a function of time in geological history. The longer geological time leads to more ^87^Sr accumulation and higher ^87^Sr/^86^Sr ration. The ^87^Sr/^86^Sr ration is not fractionated by geochemical or evaporation processes and thus retains a fingerprint of its source^21,22^. Samples of dolomite, anhydrite, polyhalite, and the PPD from salt-bearing strata were collected for strontium isotope test. The ^87^Sr/^86^Sr rations of samples, ranging from 0.70816 to 0.70837, are not significantly different. The ^87^Sr/^86^Sr rations of polyhalite, the PPD, and anhydrite increase with depth, while this increase trend is not obvious for dolomite (Fig. 7). Another strontium isotopic test was carried out on the manually selected halite and the polyhalite from the PPD at the depth of 3,467.5 m in Well ZK601. The test results are basically the same, with the data 0.708160 and 0.708161 respectively, indicating the same source.

**Figure 8:**
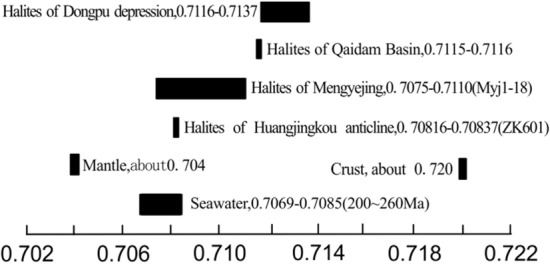
Range of ^87^Sr/^86^Sr rations from different strontium sources (mantle and lithosphere data sourced from Kelts^29^, seawater data from Burke^29^, Hess et al.^31^, halite samples in Qaidam Basin and Dongpu Depression are representative of the Land sources, from Tan^32^ and Shi^33^, Mengyejing data from Zheng et al.^34^).

The strontium isotopic data of samples were compared with those of Triassic sea water, mantle, crust and other typical domestic rock salt samples (Fig. 8). The strontium isotopic data of the salt bearing strata of Huangjinkou anticline are completely within the range of strontium isotopic data of the Triassic sea water and are far lower than those of the continental rock salt of Qaidam Basin and Dongpu Depression, which shows the marine source of the samples.

## Discussions

There are three main genesis model of polyhalite in Sichuan Basin: primary sedimentary genesis (the K^+^ and Mg^2+^ rich solution supplied by exotic Ca^2+^ or vice versa), penecontemporaneous metasomatism (the pre deposited gypsum or anhydrite replaced by further enriched brine rich in K^+^ and Mg^2+^), epigenetic metasomatism (gypsum or anhydrite replaced by brine rich in K^+^ and Mg^2+^)^23–25^.

According to the observation and analysis of core samples, the Triassic evaporite in Northeast Sichuan Basin is simply dominated by anhydrite, halite, polyhalite and magnesite. There are two main forms of polyhalite occurrence: layered, stratoid, lenticular polyhalite coexisting with anhydrite, and agglomerate, granular, dispersed polyhalite coexisting with halite. This paper mainly focuses on the latter—the PPD. Under the microscope, the polyhalites in the PPD are subhedral-xenomorphic granular, tabular, striped and do not coexist with anhydrite, which rules out the third genesis model (anhydrite replaced by brine rich in K^+^ and Mg^2+^).

In the process of evaporation, concentration and crystallization of seawater, bromine is mainly concentrated in the solution without forming a single mineral. However, due to the similar chemical properties of bromine and chloride ions, bromine will enter the chloride minerals in the form of isomorphism. In addition, the concentration of Br^−^ increases with the increase of the solution concentration, and the concentration of Br^−^ in the late-crystallized chloride is higher than that in the early-crystallized chloride.

Cheng et al. proposed that the Br^−^ × 10^–3^/Cl^−^ is 0.11 when salt minerals precipitate after the evaporation and concentration of seawater, about 0.31 when the sylvite precipitates, and 0.45 when the carnallite crystallizes and precipitates^26^. The chemical analysis results of halite samples in the study area shows that the average value of Br^−^ × 10^–3^/Cl^−^ corresponds to the stage of sylvite precipitation, and the value of Br^−^ × 10^–3^/Cl^−^ of some samples corresponds to the stage of carnallite precipitation.

The REE content and distribution pattern can reflect the deposition environment of samples. The samples have relatively low total amount of REE and distinct REE differentiation, moderate LREE fractionation, inapparent HREE fractionation, and slight negative anomaly of Ce. This indicates that the Early-Middle Triassic deposition of the Huangjinkou anticline was in deep-water reduction environment and was rarely affected by terrigenous materials. The samples have distinct REE differentiation, enriched LREE, relatively deficient HREE, and the consistence with seawater in terms of REE distribution pattern, which indicates that the sediment source of the salt bearing strata should be marine. The medium loss of δEu indicates that there is no hydrothermal fluid action during deposition and diagenesis.

According to the previous research data, during the salt forming process of the fourth and fifth member of Jialingjiang Formation–the first member of Leikoupo Formation (T_1_*j*^4 + 5^-T_2_*l*^1^) in the study area, the platform was still connected with the sea water from time to time in the course of regression^27^.

According to the Br^−^ × 10^–3^/Cl^−^ analysis of the PPD in the study area, the paleo-brine could make the deposition of potassium magnesium salt happen. However, in the evaporite minerals, the interbedding of anhydrite, halite, polyhalite and magnesite is common. This indicates that there was dilution by seawater intrusion in the salt forming process. In addition, it is generally believed that the formation of magnesite is related to seawater intrusion into the salt lake, and magnesite is generally contained in the evaporite in the study area, which further indicates that seawater intrusion occurred during the evaporation and concentration of paleo-brine.

Based on the above analysis, it is considered that the formation process of the PPD is as follows: in the stage of salt mineral deposition, the ancient seawater was further evaporated and concentrated. When the stage of sylvite deposition was not completely reached, the seawater rich in Ca^2+^ repeatedly intruded and mixed with seawater rich in K^+^ and Mg^2+^, and the polyhalite precipitated. Therefore, the PPD are primary deposits, which were structurally reformed later.

## Conclusions

The PPD in Puguang area of Northeast Sichuan Basin was developed in the salt forming period of the fifth member of Jialingjiang Formation—the first submember of the first member of Leikoupo Formation (T_1_*j*^5^-T_2_*l*^1–1^). The completely salt bearing profile is composed of carbonate rock, sulfate rock, chloride rock, sulfate rock and carbonate rock from the bottom to the top. In PPD, the polyhalite is agglomerate, granular, dispersed and thin-layered, which is beneficial to water-soluble mining.

The PPD was formed before the complete deposition of sylvite. The polyhalite precipitated after the repeatedly intruded Ca^2+^ rich seawater mixed with seawater rich in K^+^ and Mg^2+^. Under the microscope, the polyhalites are subhedral-xenomorphic granular, tabular, and striped. PPD are primary deposits without metasomatism.

In Puguang area, Northeast Sichuan Basin, the PPD is thickly deposited, widely distributed and well preserved. The thickness distribution changes obviously along the anticline trend. The characteristics above show that the PPD is controlled by the ancient evaporation center and structure. The discovery of PPD has activated the polyhalite which has long been considered as a deep useless ore, and PPD has become a large-scale high-quality potassium sulfate (K_2_SO_4_–MgSO_4_) type potassium ore that can be economically utilized. It has great potential economic value and is expected to become a new strategic base of large-scale marine solid potassium ore in China. The PDD of the Triassic in Sichuan Basin will also become one of the main directions of marine potassium exploration in the future.

### Sample collection and test

In this paper, core samples of Triassic PPD were collected from the potash exploration wells in Huangjinkou anticline. The samples were analyzed for petrology and mineralogy, using X-ray powder diffraction, chemical analysis, REEs analysis and strontium isotope test.

The X-ray powder diffraction was completed in the Research Center of Oil and Gas Resources, Northwest Institute of Ecological Environment and Resources, Chinese Academy of Sciences. The REEs test was carried out in Aoshi Analysis and Test (Guangzhou) Co., Ltd., whose laboratory has the test qualification, many years of experimental experience, and the analysis results are reliable.

Method ME-MS61 was used for LA, CE and Y. The results were obtained by four-acid digestion and mass spectrometer based quantitative analysis. Method ME-MS81 was used for other elements. The results were obtained by LiBO_2_ melting and mass spectrometer based quantitative analysis. MC-ICP-MS (multi-collector inductively coupled plasma mass spectrometer) was used for strontium isotope test. Chemical pretreatment and mass spectrometry was completed in Nanjing FocuMS Technology Co. Ltd. After the rock powder was digested by high-pressure sealed dissolution pellet, Sr was separated from the digestion solution by cation strontium specific resin. In the determination of Sr isotope ratio, ^86^Sr/^88^Sr = 0.1194 was used to calibrate the mass fractionation of the instrument. NIST SRM 987, an international standard for Sr isotopes, was used as an external standard to calibrate the drift of the instrument.
